# 2-(2*H*-Indazol-2-yl)-1-phenyl­ethanone

**DOI:** 10.1107/S1600536812051811

**Published:** 2013-01-04

**Authors:** Özden Özel Güven, Gökhan Türk, Philip D. F. Adler, Simon J. Coles, Tuncer Hökelek

**Affiliations:** a Department of Chemistry, Bülent Ecevit University, 67100 Zonguldak, Turkey; b Department of Chemistry, Southampton University, SO17 1BJ Southampton, England; c Department of Physics, Hacettepe University, 06800 Beytepe, Ankara, Turkey

## Abstract

The asymmetric unit of the title compound, C_15_H_12_N_2_O, contains two independent mol­ecules with different conformations, the phenyl ring and indazole mean plane in the two mol­ecules forming dihedral angles of 50.82 (5) and 89.29 (6)°. In the crystal, weak C—H⋯O and C–H⋯N hydrogen bonds and C—H⋯π inter­actions consolidate the packing.

## Related literature

For general background to the biological activity of indazole derivatives, see: Lebouvier *et al.* (2007 ▶); Maggio *et al.* (2011 ▶); Park *et al.* (2007 ▶); Plescia *et al.* (2010 ▶); Raffa *et al.* (2009 ▶). For related structures, see: Gerpe *et al.* (2007 ▶); Özel Güven *et al.* (2008*a* ▶,*b* ▶); Raffa *et al.* (2009 ▶).
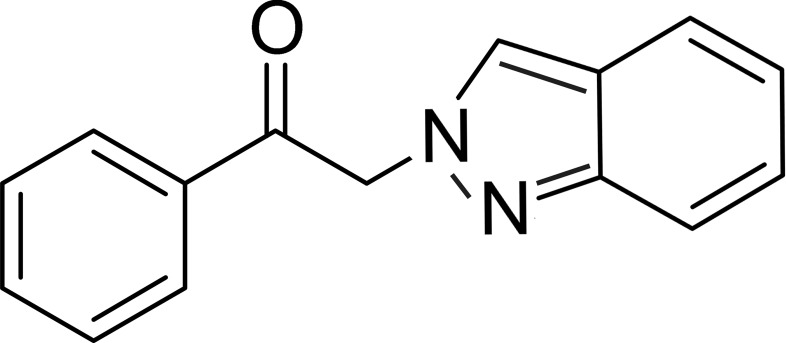


## Experimental

### 

#### Crystal data

C_15_H_12_N_2_O
*M* *_r_* = 236.27
Monoclinic, 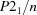

*a* = 9.4408 (3) Å
*b* = 17.9636 (5) Å
*c* = 13.9415 (4) Å
β = 99.247 (4)°
*V* = 2333.62 (12) Å^3^
*Z* = 8
Mo *K*α radiation
μ = 0.09 mm^−1^
*T* = 100 K
0.20 × 0.20 × 0.20 mm

#### Data collection

Rigaku Saturn724+ diffractometer
23377 measured reflections
5349 independent reflections
3346 reflections with *I* > 2σ(*I*)
*R*_int_ = 0.084
3 standard reflections every 2 min intensity decay: 1%

#### Refinement

*R*[*F* ^2^ > 2σ(*F* ^2^)] = 0.057
*wR*(*F* ^2^) = 0.129
*S* = 1.02
5349 reflections
325 parameters
H-atom parameters constrained
Δρ_max_ = 0.30 e Å^−3^
Δρ_min_ = −0.24 e Å^−3^

### 

Data collection: *CrystalClear-SM Expert* (Rigaku, 2011 ▶); cell refinement: *CrystalClear-SM Expert*; data reduction: *CrystalClear-SM Expert*; program(s) used to solve structure: *SHELXS97* (Sheldrick, 2008 ▶); program(s) used to refine structure: *SHELXL97* (Sheldrick, 2008 ▶); molecular graphics: *ORTEP-3 for Windows* (Farrugia, 2012 ▶); software used to prepare material for publication: *WinGX* publication routines (Farrugia, 2012 ▶) and *PLATON* (Spek, 2009 ▶).

## Supplementary Material

Crystal structure: contains datablock(s) I, global. DOI: 10.1107/S1600536812051811/cv5372sup1.cif

Structure factors: contains datablock(s) I. DOI: 10.1107/S1600536812051811/cv5372Isup2.hkl

Additional supplementary materials:  crystallographic information; 3D view; checkCIF report

## Figures and Tables

**Table 1:** Hydrogen-bond geometry (Å, °) *Cg*1 and *Cg*2 are the centroids of the N1*A*/N2*A*/C9*A*/C10*A*/C15*A* and C10*B*–C15*B* rings, respectively.

*D*—H⋯*A*	*D*—H	H⋯*A*	*D*⋯*A*	*D*—H⋯*A*
C6*A*—H6*A*⋯O1*B* ^i^	0.93	2.53	3.418 (2)	159
C8*A*—H81⋯N2*A* ^ii^	0.97	2.57	3.519 (3)	164
C8*A*—H82⋯O1*B* ^iii^	0.97	2.41	3.176 (2)	135
C9*B*—H9*B*⋯*Cg*1^iv^	0.93	2.86	3.460 (2)	123
C3*B*—H3*B*⋯*Cg*2^v^	0.93	2.60	3.433 (2)	149

Symmetry codes: (i) 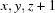; (ii) 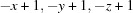; (iii) 
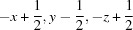
; (iv) 
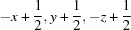
; (v) 
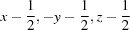
.

**Fig. 1.**
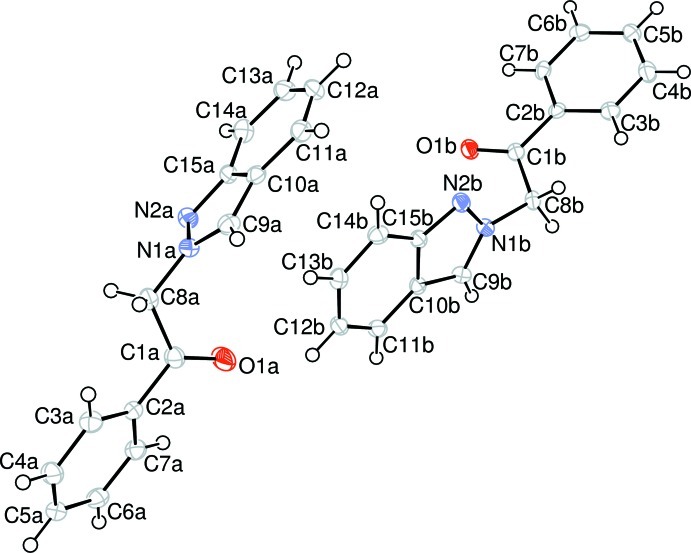
Two independent molecules in (I) with the atom-numbering scheme. Displacement ellipsoids are drawn at the 50% probability level.

## supplementary crystallographic information

### Comment

Azole compounds have important biological activities. Some indazole derivatives have been known as antifungal (Lebouvier *et al.*, 2007; Park *et al.*, 2007) and antiproliferative agents (Raffa *et al.*, 2009; Plescia *et al.*, 2010; Maggio *et al.*, 2011) and crystal structures have been reported (Gerpe *et al.*, 2007; Raffa *et al.*, 2009). Crystal structures of ketones similar to the titled compound having benzimidazole ring (Özel Güven *et al.*, 2008*a*) and 1,2,4-triazole ring (Özel Güven *et al.*, 2008*b*) have been reported. Now we report the crystal structure of the title indazole derivative, (I).

The asymmetric unit of (I) contains two crystallographically independent molecules (Fig. 1), in which the bond lengths and angles are generally within normal ranges. The indazole [B (N1A/N2A/C9A-C15A) and B' (N1B/N2B/C9B-C15B)] ring systems are approximately planar with maximum deviations of -0.013 (2)Å (for atom C13A) and -0.025 (2)Å (for atom C12B), respectively. Their mean planes are oriented with respect to the phenyl [A (C2A-C7A) and A' (C2B-C7B)] rings at dihedral angles of A/B = 50.82 (5) and A'/B' = 89.29 (6) °. The dihedral angles between the rings A, A' and B, B' are A/A' = 78.52 (7) and B/B' = 62.38 (5) °. Atoms C8A and C8B are -0.048 (2) and -0.088 (2) Å away from the corresponding indazole ring planes, while atoms C1A, O1A and C1B, O1B are -0.022 (2), 0.516 (2) Å and -0.024 (2), 0.039 (1) Å away from the corresponding phenyl ring planes.

In the crystal structure, weak intermolecular C—H···O and C—H···N hydrogen bonds, and C—H···π interactions (Table 1) consolidate the packing.

### Experimental

The title compound, (I), was synthesized by the reaction of 2-bromo-1-phenylethanone with 1*H*-imidazole. A mixture of 2-bromo-1-phenylethanone (0.842 g, 4.232 mmol) and 1*H*-imidazole (1 g, 8.465 mmol) was refluxed in toluene (40 ml) for 9 h. After evaporation of the solvent, the formed precipitate was purified by column chromatography using hexane-ethylacetate (5:1) mixture, and then crystallized from chloroform to obtain colorless crystals suitable for X-ray analysis (yield; 0.22 g, 22%).

### Refinement

H atoms were positioned geometrically with C—H = 0.93 and 0.97 Å for aromatic and methylene H, respectively, and constrained to ride on their parent atoms, with *U*_iso_(H) = 1.2 *U*_eq_(C).

### Crystal data

**Table:**

C_15_H_12_N_2_O	*F*(000) = 992
*M**_r_* = 236.27	*D*_x_ = 1.345 Mg m^−^^3^
Monoclinic, *P*2_1_/*n*	Mo *K*α radiation, λ = 0.71073 Å
Hall symbol: -P 2yn	Cell parameters from 15539 reflections
*a* = 9.4408 (3) Å	θ = 3.1–27.5°
*b* = 17.9636 (5) Å	µ = 0.09 mm^−^^1^
*c* = 13.9415 (4) Å	*T* = 100 K
β = 99.247 (4)°	Prism, colorless
*V* = 2333.62 (12) Å^3^	0.20 × 0.20 × 0.20 mm
*Z* = 8	

### Data collection

**Table:**

Rigaku Saturn724+ diffractometer	*R*_int_ = 0.084
Radiation source: fine-focus sealed tube	θ_max_ = 27.5°, θ_min_ = 3.1°
Graphite monochromator	*h* = −12→12
profile data from ω–scans	*k* = −21→23
23377 measured reflections	*l* = −18→18
5349 independent reflections	3 standard reflections every 2 min
3346 reflections with *I* > 2σ(*I*)	intensity decay: 1%

### Refinement

**Table:**

Refinement on *F*^2^	Primary atom site location: structure-invariant direct methods
Least-squares matrix: full	Secondary atom site location: difference Fourier map
*R*[*F*^2^ > 2σ(*F*^2^)] = 0.057	Hydrogen site location: inferred from neighbouring sites
*wR*(*F*^2^) = 0.129	H-atom parameters constrained
*S* = 1.02	*w* = 1/[σ^2^(*F*_o_^2^) + (0.0558*P*)^2^] where *P* = (*F*_o_^2^ + 2*F*_c_^2^)/3
5349 reflections	(Δ/σ)_max_ < 0.001
325 parameters	Δρ_max_ = 0.30 e Å^−^^3^
0 restraints	Δρ_min_ = −0.24 e Å^−^^3^

### Special details

**Table:**

Geometry. All esds (except the esd in the dihedral angle between two l.s. planes) are estimated using the full covariance matrix. The cell esds are taken into account individually in the estimation of esds in distances, angles and torsion angles; correlations between esds in cell parameters are only used when they are defined by crystal symmetry. An approximate (isotropic) treatment of cell esds is used for estimating esds involving l.s. planes.
Refinement. Refinement of F^2^ against ALL reflections. The weighted R-factor wR and goodness of fit S are based on F^2^, conventional R-factors R are based on F, with F set to zero for negative F^2^. The threshold expression of F^2^ > 2sigma(F^2^) is used only for calculating R-factors(gt) etc. and is not relevant to the choice of reflections for refinement. R-factors based on F^2^ are statistically about twice as large as those based on F, and R- factors based on ALL data will be even larger.

### Fractional atomic coordinates and isotropic or equivalent isotropic displacement parameters (Å^2^)

**Table:**

	*x*	*y*	*z*	*U*_iso_*/*U*_eq_	
O1A	0.14439 (19)	0.58400 (8)	0.50109 (10)	0.0407 (5)	
N1A	0.26416 (18)	0.46418 (9)	0.42124 (11)	0.0201 (4)	
N2A	0.36687 (18)	0.49886 (9)	0.37948 (11)	0.0215 (4)	
C1A	0.2002 (2)	0.53784 (11)	0.55820 (14)	0.0229 (5)	
C2A	0.2122 (2)	0.54710 (10)	0.66507 (13)	0.0184 (4)	
C3A	0.2094 (2)	0.48688 (11)	0.72716 (14)	0.0229 (5)	
H3A	0.1993	0.4389	0.7020	0.028*	
C4A	0.2214 (2)	0.49816 (11)	0.82645 (14)	0.0255 (5)	
H4A	0.2173	0.4579	0.8678	0.031*	
C5A	0.2394 (2)	0.56951 (11)	0.86395 (14)	0.0240 (5)	
H5A	0.2482	0.5770	0.9306	0.029*	
C6A	0.2443 (2)	0.62952 (11)	0.80282 (14)	0.0239 (5)	
H6A	0.2583	0.6773	0.8283	0.029*	
C7A	0.2285 (2)	0.61847 (11)	0.70379 (14)	0.0225 (5)	
H7A	0.2286	0.6591	0.6625	0.027*	
C8A	0.2702 (2)	0.46866 (11)	0.52526 (13)	0.0236 (5)	
H81	0.3699	0.4676	0.5561	0.028*	
H82	0.2234	0.4252	0.5470	0.028*	
C9A	0.1661 (2)	0.42712 (11)	0.35829 (13)	0.0221 (5)	
H9A	0.0880	0.4003	0.3729	0.026*	
C10A	0.2049 (2)	0.43697 (10)	0.26711 (13)	0.0196 (4)	
C11A	0.1489 (2)	0.41293 (11)	0.17170 (14)	0.0236 (5)	
H11A	0.0663	0.3840	0.1595	0.028*	
C12A	0.2199 (2)	0.43368 (11)	0.09856 (14)	0.0255 (5)	
H12A	0.1849	0.4187	0.0354	0.031*	
C13A	0.3461 (2)	0.47761 (11)	0.11623 (14)	0.0263 (5)	
H13A	0.3922	0.4900	0.0643	0.032*	
C14A	0.4020 (2)	0.50222 (11)	0.20685 (14)	0.0246 (5)	
H14A	0.4844	0.5314	0.2175	0.030*	
C15A	0.3296 (2)	0.48162 (10)	0.28413 (13)	0.0191 (4)	
O1B	0.19943 (15)	0.80396 (7)	−0.10890 (9)	0.0229 (3)	
N1B	−0.01229 (18)	0.77510 (9)	−0.00267 (11)	0.0195 (4)	
N2B	0.00706 (18)	0.70086 (9)	0.01224 (11)	0.0202 (4)	
C1B	0.0784 (2)	0.80142 (10)	−0.15520 (13)	0.0187 (4)	
C2B	0.0524 (2)	0.80064 (10)	−0.26292 (13)	0.0177 (4)	
C3B	−0.0848 (2)	0.79327 (12)	−0.31651 (14)	0.0251 (5)	
H3B	−0.1635	0.7880	−0.2846	0.030*	
C4B	−0.1043 (2)	0.79378 (13)	−0.41696 (14)	0.0312 (5)	
H4B	−0.1960	0.7887	−0.4525	0.037*	
C5B	0.0122 (2)	0.80181 (12)	−0.46459 (14)	0.0267 (5)	
H5B	−0.0016	0.8031	−0.5321	0.032*	
C6B	0.1488 (2)	0.80796 (11)	−0.41253 (13)	0.0220 (5)	
H6B	0.2271	0.8123	−0.4450	0.026*	
C7B	0.1694 (2)	0.80764 (10)	−0.31214 (13)	0.0193 (4)	
H7B	0.2616	0.8121	−0.2772	0.023*	
C8B	−0.0503 (2)	0.80013 (12)	−0.10227 (13)	0.0220 (5)	
H83	−0.0910	0.8497	−0.1028	0.026*	
H84	−0.1230	0.7673	−0.1364	0.026*	
C9B	0.0048 (2)	0.81611 (11)	0.07851 (13)	0.0200 (5)	
H9B	−0.0025	0.8676	0.0826	0.024*	
C10B	0.0357 (2)	0.76636 (11)	0.15582 (13)	0.0173 (4)	
C11B	0.0610 (2)	0.77194 (11)	0.25834 (13)	0.0209 (5)	
H11B	0.0627	0.8179	0.2891	0.025*	
C12B	0.0828 (2)	0.70741 (11)	0.31061 (13)	0.0215 (5)	
H12B	0.0969	0.7098	0.3781	0.026*	
C13B	0.0847 (2)	0.63700 (11)	0.26516 (14)	0.0218 (5)	
H13B	0.1013	0.5946	0.3035	0.026*	
C14B	0.0627 (2)	0.62993 (11)	0.16617 (14)	0.0214 (5)	
H14B	0.0651	0.5836	0.1367	0.026*	
C15B	0.0363 (2)	0.69534 (11)	0.11057 (13)	0.0179 (4)	

### Atomic displacement parameters (Å^2^)

**Table:**

	*U*^11^	*U*^22^	*U*^33^	*U*^12^	*U*^13^	*U*^23^
O1A	0.0671 (13)	0.0324 (9)	0.0216 (8)	0.0229 (9)	0.0043 (8)	0.0037 (7)
N1A	0.0220 (10)	0.0202 (9)	0.0183 (8)	0.0025 (8)	0.0036 (7)	−0.0013 (7)
N2A	0.0221 (10)	0.0222 (9)	0.0195 (9)	−0.0008 (8)	0.0015 (7)	−0.0010 (7)
C1A	0.0273 (12)	0.0217 (11)	0.0200 (10)	0.0018 (9)	0.0043 (9)	0.0035 (9)
C2A	0.0168 (11)	0.0197 (11)	0.0188 (10)	0.0016 (8)	0.0034 (8)	0.0002 (8)
C3A	0.0257 (12)	0.0185 (11)	0.0250 (11)	−0.0011 (9)	0.0052 (9)	−0.0012 (9)
C4A	0.0324 (13)	0.0230 (11)	0.0221 (10)	0.0034 (10)	0.0071 (9)	0.0051 (9)
C5A	0.0244 (12)	0.0292 (12)	0.0189 (10)	0.0055 (10)	0.0050 (9)	0.0000 (9)
C6A	0.0233 (12)	0.0211 (11)	0.0276 (11)	0.0016 (9)	0.0051 (9)	−0.0043 (9)
C7A	0.0260 (12)	0.0182 (11)	0.0241 (11)	0.0026 (9)	0.0069 (9)	0.0032 (8)
C8A	0.0270 (12)	0.0250 (11)	0.0177 (10)	0.0042 (10)	0.0001 (9)	0.0014 (9)
C9A	0.0202 (11)	0.0205 (11)	0.0257 (11)	−0.0006 (9)	0.0046 (9)	−0.0025 (9)
C10A	0.0192 (11)	0.0169 (10)	0.0223 (10)	0.0031 (9)	0.0021 (8)	−0.0028 (8)
C11A	0.0218 (12)	0.0208 (11)	0.0265 (11)	0.0000 (9)	−0.0009 (9)	−0.0041 (9)
C12A	0.0306 (13)	0.0267 (12)	0.0176 (10)	0.0044 (10)	−0.0012 (9)	−0.0048 (9)
C13A	0.0301 (13)	0.0281 (12)	0.0218 (11)	0.0043 (10)	0.0072 (9)	0.0019 (9)
C14A	0.0230 (12)	0.0230 (11)	0.0268 (11)	−0.0020 (9)	0.0009 (9)	0.0017 (9)
C15A	0.0213 (11)	0.0176 (10)	0.0174 (10)	0.0037 (8)	0.0002 (8)	−0.0010 (8)
O1B	0.0217 (8)	0.0265 (8)	0.0193 (7)	−0.0010 (6)	0.0002 (6)	−0.0010 (6)
N1B	0.0206 (10)	0.0218 (9)	0.0162 (8)	0.0006 (7)	0.0030 (7)	−0.0001 (7)
N2B	0.0209 (10)	0.0200 (9)	0.0189 (8)	0.0002 (7)	0.0003 (7)	−0.0013 (7)
C1B	0.0231 (12)	0.0121 (10)	0.0198 (10)	0.0015 (9)	−0.0002 (9)	−0.0011 (8)
C2B	0.0197 (11)	0.0151 (10)	0.0187 (9)	0.0038 (8)	0.0041 (8)	0.0001 (8)
C3B	0.0205 (12)	0.0347 (13)	0.0211 (10)	0.0012 (10)	0.0063 (9)	−0.0017 (9)
C4B	0.0217 (13)	0.0483 (15)	0.0224 (11)	0.0012 (11)	−0.0005 (9)	−0.0044 (10)
C5B	0.0301 (13)	0.0343 (13)	0.0156 (10)	0.0081 (10)	0.0032 (9)	0.0019 (9)
C6B	0.0269 (13)	0.0205 (11)	0.0210 (10)	0.0015 (9)	0.0108 (9)	0.0035 (8)
C7B	0.0174 (11)	0.0150 (10)	0.0243 (10)	0.0007 (8)	−0.0006 (9)	0.0002 (8)
C8B	0.0181 (11)	0.0291 (12)	0.0173 (10)	0.0027 (9)	−0.0010 (8)	0.0005 (9)
C9B	0.0219 (12)	0.0194 (11)	0.0191 (10)	−0.0005 (9)	0.0048 (9)	−0.0021 (8)
C10B	0.0130 (10)	0.0217 (11)	0.0170 (10)	−0.0015 (8)	0.0023 (8)	0.0005 (8)
C11B	0.0187 (11)	0.0249 (11)	0.0197 (10)	−0.0001 (9)	0.0046 (9)	−0.0030 (9)
C12B	0.0166 (11)	0.0319 (12)	0.0158 (9)	−0.0009 (9)	0.0022 (8)	−0.0009 (9)
C13B	0.0215 (11)	0.0219 (11)	0.0216 (10)	0.0013 (9)	0.0016 (9)	0.0032 (8)
C14B	0.0215 (12)	0.0187 (11)	0.0243 (11)	−0.0001 (9)	0.0047 (9)	−0.0022 (9)
C15B	0.0133 (10)	0.0243 (11)	0.0160 (9)	−0.0019 (9)	0.0023 (8)	0.0000 (8)

### Geometric parameters (Å, º)

**Table:**

O1A—C1A	1.210 (2)	O1B—C1B	1.219 (2)
N1A—C8A	1.444 (2)	N1B—C8B	1.449 (2)
N1A—C9A	1.345 (2)	N1B—C9B	1.338 (2)
N2A—N1A	1.360 (2)	N2B—N1B	1.357 (2)
N2A—C15A	1.355 (2)	N2B—C15B	1.358 (2)
C1A—C2A	1.485 (3)	C1B—C8B	1.520 (3)
C1A—C8A	1.513 (3)	C2B—C1B	1.482 (3)
C2A—C3A	1.388 (3)	C2B—C3B	1.394 (3)
C2A—C7A	1.390 (3)	C2B—C7B	1.396 (3)
C3A—C4A	1.385 (3)	C3B—C4B	1.383 (3)
C3A—H3A	0.9300	C3B—H3B	0.9300
C4A—H4A	0.9300	C4B—H4B	0.9300
C5A—C4A	1.384 (3)	C5B—C4B	1.380 (3)
C5A—C6A	1.380 (3)	C5B—H5B	0.9300
C5A—H5A	0.9300	C6B—C5B	1.379 (3)
C6A—H6A	0.9300	C6B—C7B	1.382 (2)
C7A—C6A	1.379 (3)	C6B—H6B	0.9300
C7A—H7A	0.9300	C7B—H7B	0.9300
C8A—H81	0.9700	C8B—H83	0.9700
C8A—H82	0.9700	C8B—H84	0.9700
C9A—C10A	1.389 (3)	C9B—C10B	1.395 (3)
C9A—H9A	0.9300	C9B—H9B	0.9300
C10A—C11A	1.417 (3)	C10B—C11B	1.414 (2)
C11A—H11A	0.9300	C11B—C12B	1.367 (3)
C12A—C11A	1.360 (3)	C11B—H11B	0.9300
C12A—H12A	0.9300	C12B—H12B	0.9300
C13A—C12A	1.417 (3)	C13B—C12B	1.416 (3)
C13A—C14A	1.362 (3)	C13B—C14B	1.368 (3)
C13A—H13A	0.9300	C13B—H13B	0.9300
C14A—H14A	0.9300	C14B—H14B	0.9300
C15A—C10A	1.413 (3)	C15B—C10B	1.424 (3)
C15A—C14A	1.415 (3)	C15B—C14B	1.408 (3)
			
N2A—N1A—C8A	119.24 (16)	N2B—N1B—C8B	117.27 (15)
C9A—N1A—N2A	114.25 (15)	C9B—N1B—N2B	114.60 (15)
C9A—N1A—C8A	126.50 (17)	C9B—N1B—C8B	128.12 (17)
C15A—N2A—N1A	102.99 (15)	N1B—N2B—C15B	103.12 (15)
O1A—C1A—C2A	122.58 (18)	O1B—C1B—C2B	121.69 (18)
O1A—C1A—C8A	121.89 (17)	O1B—C1B—C8B	119.85 (16)
C2A—C1A—C8A	115.43 (16)	C2B—C1B—C8B	118.45 (17)
C3A—C2A—C1A	122.15 (17)	C3B—C2B—C1B	122.16 (18)
C3A—C2A—C7A	119.24 (17)	C3B—C2B—C7B	119.05 (17)
C7A—C2A—C1A	118.61 (17)	C7B—C2B—C1B	118.79 (18)
C2A—C3A—H3A	119.9	C2B—C3B—H3B	119.9
C4A—C3A—C2A	120.15 (19)	C4B—C3B—C2B	120.2 (2)
C4A—C3A—H3A	119.9	C4B—C3B—H3B	119.9
C3A—C4A—H4A	120.1	C3B—C4B—H4B	119.9
C5A—C4A—C3A	119.87 (19)	C5B—C4B—C3B	120.1 (2)
C5A—C4A—H4A	120.1	C5B—C4B—H4B	119.9
C4A—C5A—H5A	119.9	C4B—C5B—H5B	119.8
C6A—C5A—C4A	120.30 (18)	C6B—C5B—C4B	120.34 (18)
C6A—C5A—H5A	119.9	C6B—C5B—H5B	119.8
C5A—C6A—H6A	120.1	C5B—C6B—C7B	119.99 (19)
C7A—C6A—C5A	119.80 (19)	C5B—C6B—H6B	120.0
C7A—C6A—H6A	120.1	C7B—C6B—H6B	120.0
C2A—C7A—H7A	119.7	C2B—C7B—H7B	119.8
C6A—C7A—C2A	120.60 (18)	C6B—C7B—C2B	120.31 (19)
C6A—C7A—H7A	119.7	C6B—C7B—H7B	119.8
N1A—C8A—C1A	113.68 (16)	N1B—C8B—C1B	112.07 (16)
N1A—C8A—H81	108.8	N1B—C8B—H83	109.2
N1A—C8A—H82	108.8	N1B—C8B—H84	109.2
C1A—C8A—H81	108.8	C1B—C8B—H83	109.2
C1A—C8A—H82	108.8	C1B—C8B—H84	109.2
H81—C8A—H82	107.7	H83—C8B—H84	107.9
N1A—C9A—C10A	106.21 (18)	N1B—C9B—C10B	106.42 (17)
N1A—C9A—H9A	126.9	N1B—C9B—H9B	126.8
C10A—C9A—H9A	126.9	C10B—C9B—H9B	126.8
C9A—C10A—C11A	134.95 (19)	C9B—C10B—C11B	135.73 (18)
C9A—C10A—C15A	104.68 (17)	C9B—C10B—C15B	104.30 (16)
C15A—C10A—C11A	120.37 (18)	C11B—C10B—C15B	119.96 (17)
C12A—C11A—C10A	117.76 (19)	C10B—C11B—H11B	121.1
C12A—C11A—H11A	121.1	C12B—C11B—C10B	117.71 (18)
C10A—C11A—H11A	121.1	C12B—C11B—H11B	121.1
C11A—C12A—C13A	121.64 (18)	C11B—C12B—C13B	122.04 (17)
C11A—C12A—H12A	119.2	C11B—C12B—H12B	119.0
C13A—C12A—H12A	119.2	C13B—C12B—H12B	119.0
C12A—C13A—H13A	118.9	C12B—C13B—H13B	119.2
C14A—C13A—C12A	122.1 (2)	C14B—C13B—C12B	121.56 (18)
C14A—C13A—H13A	118.9	C14B—C13B—H13B	119.2
C13A—C14A—C15A	117.3 (2)	C13B—C14B—C15B	117.57 (18)
C13A—C14A—H14A	121.3	C13B—C14B—H14B	121.2
C15A—C14A—H14A	121.3	C15B—C14B—H14B	121.2
N2A—C15A—C10A	111.87 (17)	N2B—C15B—C10B	111.55 (17)
N2A—C15A—C14A	127.32 (19)	N2B—C15B—C14B	127.32 (18)
C10A—C15A—C14A	120.80 (17)	C14B—C15B—C10B	121.12 (16)
			
N2A—N1A—C8A—C1A	85.3 (2)	N2B—N1B—C8B—C1B	−77.3 (2)
C9A—N1A—C8A—C1A	−96.0 (2)	C9B—N1B—C8B—C1B	103.7 (2)
N2A—N1A—C9A—C10A	0.2 (2)	N2B—N1B—C9B—C10B	−1.2 (2)
C8A—N1A—C9A—C10A	−178.57 (17)	C8B—N1B—C9B—C10B	177.81 (18)
C15A—N2A—N1A—C8A	178.61 (16)	C15B—N2B—N1B—C8B	−178.14 (16)
C15A—N2A—N1A—C9A	−0.3 (2)	C15B—N2B—N1B—C9B	1.0 (2)
N1A—N2A—C15A—C10A	0.2 (2)	N1B—N2B—C15B—C10B	−0.4 (2)
N1A—N2A—C15A—C14A	−178.65 (19)	N1B—N2B—C15B—C14B	178.66 (19)
O1A—C1A—C2A—C3A	148.4 (2)	O1B—C1B—C8B—N1B	−21.2 (3)
O1A—C1A—C2A—C7A	−32.3 (3)	C2B—C1B—C8B—N1B	159.71 (16)
C8A—C1A—C2A—C3A	−35.1 (3)	C3B—C2B—C1B—O1B	176.09 (18)
C8A—C1A—C2A—C7A	144.24 (19)	C3B—C2B—C1B—C8B	−4.8 (3)
O1A—C1A—C8A—N1A	0.4 (3)	C7B—C2B—C1B—O1B	−4.0 (3)
C2A—C1A—C8A—N1A	−176.21 (17)	C7B—C2B—C1B—C8B	175.09 (17)
C1A—C2A—C3A—C4A	179.82 (19)	C1B—C2B—C3B—C4B	179.16 (19)
C7A—C2A—C3A—C4A	0.5 (3)	C7B—C2B—C3B—C4B	−0.7 (3)
C1A—C2A—C7A—C6A	−178.09 (19)	C1B—C2B—C7B—C6B	−179.28 (17)
C3A—C2A—C7A—C6A	1.2 (3)	C3B—C2B—C7B—C6B	0.6 (3)
C2A—C3A—C4A—C5A	−1.4 (3)	C2B—C3B—C4B—C5B	−0.2 (3)
C6A—C5A—C4A—C3A	0.5 (3)	C6B—C5B—C4B—C3B	1.2 (3)
C4A—C5A—C6A—C7A	1.2 (3)	C7B—C6B—C5B—C4B	−1.3 (3)
C2A—C7A—C6A—C5A	−2.1 (3)	C5B—C6B—C7B—C2B	0.4 (3)
N1A—C9A—C10A—C11A	179.7 (2)	N1B—C9B—C10B—C11B	−177.8 (2)
N1A—C9A—C10A—C15A	−0.1 (2)	N1B—C9B—C10B—C15B	0.8 (2)
C9A—C10A—C11A—C12A	−179.0 (2)	C9B—C10B—C11B—C12B	177.4 (2)
C15A—C10A—C11A—C12A	0.6 (3)	C15B—C10B—C11B—C12B	−1.1 (3)
C13A—C12A—C11A—C10A	0.2 (3)	C10B—C11B—C12B—C13B	1.8 (3)
C14A—C13A—C12A—C11A	−0.9 (3)	C14B—C13B—C12B—C11B	−0.9 (3)
C12A—C13A—C14A—C15A	0.6 (3)	C12B—C13B—C14B—C15B	−0.7 (3)
N2A—C15A—C10A—C9A	−0.1 (2)	N2B—C15B—C10B—C9B	−0.3 (2)
N2A—C15A—C10A—C11A	−179.87 (17)	N2B—C15B—C10B—C11B	178.66 (17)
C14A—C15A—C10A—C9A	178.84 (18)	C14B—C15B—C10B—C9B	−179.40 (18)
C14A—C15A—C10A—C11A	−0.9 (3)	C14B—C15B—C10B—C11B	−0.5 (3)
N2A—C15A—C14A—C13A	179.07 (19)	N2B—C15B—C14B—C13B	−177.60 (19)
C10A—C15A—C14A—C13A	0.3 (3)	C10B—C15B—C14B—C13B	1.4 (3)

### Hydrogen-bond geometry (Å, º)

Cg1 and Cg2 are the centroids of the N1A/N2A/C9A/C10A/C15A and C10B–C15B rings, respectively.

**Table:**

*D*—H···*A*	*D*—H	H···*A*	*D*···*A*	*D*—H···*A*
C6*A*—H6*A*···O1*B*^i^	0.93	2.53	3.418 (2)	159
C8*A*—H81···N2*A*^ii^	0.97	2.57	3.519 (3)	164
C8*A*—H82···O1*B*^iii^	0.97	2.41	3.176 (2)	135
C9*B*—H9*B*···*Cg*1^iv^	0.93	2.86	3.460 (2)	123
C3*B*—H3*B*···*Cg*2^v^	0.93	2.60	3.433 (2)	149

Symmetry codes: (i) *x*, *y*, *z*+1; (ii) −*x*+1, −*y*+1, −*z*+1; (iii) −*x*+1/2, *y*−1/2, −*z*+1/2; (iv) −*x*+1/2, *y*+1/2, −*z*+1/2; (v) *x*−1/2, −*y*−1/2, *z*−1/2.
